# The association between thyroid echogenicity and thyroid function in pediatric and adolescent Hashimoto's thyroiditis

**DOI:** 10.1097/MD.0000000000015055

**Published:** 2019-04-05

**Authors:** Sun Hye Jeong, Hyun Sook Hong, Ji Ye Lee

**Affiliations:** a Department of Radiology, Soonchunhyang University Bucheon Hospital, Bucheon; b Department of Radiology, Eulji Medical center, 68 gil Hangulbisuk-ro, No won-gu, Seoul, Korea.

**Keywords:** children, Hashimoto's thyroiditis, thyroiditis, ultrasonography

## Abstract

In this study, we evaluated the association between thyroid echogenicity on ultrasonography (US) and thyroid function in pediatric and adolescent Hashimoto's thyroiditis (HT) patients.

In 86 pediatric and adolescent HT patients, the association between echogenicity and thyroid function and microsomal autoantibody status was evaluated. Among patients with overt hypothyroidism, 89.2% (33/37) showed a US grade of 3 or 4. All of the patients at grade 4 presented with overt hypothyroidism. In contrast, 97.8% (44/49) of the patients with subclinical hypothyroidism or euthyroidism showed grades 1 or 2. Patients with increased thyroid-stimulating hormone titer also tended to have increased US grades (*P* < .001). In contrast, free thyroxine levels were significantly decreased with increasing US grade (*P* < .001).

In conclusion, patients with higher US grades had decreased thyroid function (*P* < .001).

## 1 Introduction

Autoimmune thyroiditis (AIT) comprises a series of interrelated conditions including Graves’ disease and Hashimoto's thyroiditis. It is a chronic disorder with a prevalence of 1.3% to 9.6% in children and adolescents, and is characterized by the loss of tolerance to self-thyroid antigens.^[1]^ It is also the most common cause of thyroid dysfunction in children and adolescents, and is responsible for the majority of cases of acquired hypothyroidism with or without a goiter.^[2,3]^

Previous studies have reported ultrasonography (US) findings of thyroiditis and the association between echogenicity of the thyroid gland and thyroid function. Decreased echogenicity of the thyroid gland is associated with overt hypothyroidism.^[4]^ Changes in echogenicity have been demonstrated in subclinical hypothyroidism.^[5–10]^ Decreased echogenicity or irregularity in the echo pattern during a thyroid US in patients with elevated thyroid-stimulating hormone (TSH) could possibly be taken as early signs of thyroid failure. Thyroid US in subclinical hypothyroidism has a significant predictive value for treatment outcome that is comparable to other well-known prognostic parameters, such as antithyroid peroxidase antibody (TPOAb) or high TSH levels; this is because subclinical thyroid dysfunction tends to develop into overt thyroid dysfunction.^[11]^ Progression to overt hypothyroidism, with the associated adverse effects on lipid metabolism and the cardiovascular system, are major concerns for patients diagnosed with subclinical hypothyroidism. The risk of progression to overt hypothyroidism in subclinical hypothyroid patients is known to be higher in those with underlying thyroid disease.^[12]^ However, it is difficult to predict the risk of natural disease progression to a more severe form of thyroid dysfunction. There is no consensus on the clinical parameters associated with prognosis for this mild form of thyroid dysfunction, although elevation of serum TPOAb and decreased or heterogeneous echogenicity on US are commonly observed. Thyroid US in conjunction with TPOAb assay for the initial assessment of a patient with subclinical hypothyroidism appear to be more helpful than TPOAb alone for predicting the clinical course and establishing a treatment plan, especially in cases with mild elevation of serum TSH.^[13]^

To the best of our knowledge, no studies have evaluated the relationship between thyroid US echogenicity and thyroid function in pediatric and adolescent patients. Therefore, this study retrospectively evaluated echogenicity in US scans and thyroid function in pediatric and adolescent patients with Hashimoto's thyroiditis (HT).

## 2 Materials and methods

### 2.1 Patients and thyroid function analysis

This retrospective study was approved by our Institutional Review Board and the requirement for informed patient consent was waived. The study was performed in accordance with the ethical standards laid down in the 1964 Declaration of Helsinki and its later amendments.

At our institution, 117 patients were diagnosed with pediatric or adolescent HT between February 2006 and July 2016. Of these, 88 had undergone thyroid US. Two patients were excluded because the US scans showed diffusely scattered microcalcifications, making a true evaluation of thyroid echogenicity difficult. Ultimately, 86 patients were enrolled in this study. Laboratory data obtained from the medical records were evaluated retrospectively, including the serum levels of free triiodothyronine (T3), free thyroxine (FT4), TSH, antimicrosomal antibody, and antithyroglobulin autoantibodies (TGAbs). Radioimmunoassays were used for the laboratory examinations (RIA-gnost T3 & RIA-gnost TSH [Cisbio Bioassays, Codolet, France], FT4 RIA kit [Beckman Coulter, Brea, CA], Thyroid peroxidase antibody direct assay kit and thyroglobulin antibody direct assay kit [RSR LIMITED, Cardiff, United Kingdom]) and were performed on the day closest to the US examination date. Diagnosis of HT was based on the presence of autoantibodies (TPOAbs and TGAbs). Some patients also underwent fine needle aspiration biopsy (n = 1) or core biopsy (n = 2) for lymphocytic thyroiditis, or surgery due to associated nodules (n = 1 for follicular adenoma, n = 7 for papillary thyroid cancer).

Thyroid function was subdivided into overt hypothyroidism (Ia), subclinical hypothyroidism (Ib), euthyroid (II), and hyperthyroidism (III). Overt hypothyroidism was defined by decreased concentrations of T4 and elevated serum concentrations of TSH (>20 mU/L). Ib was defined as an increased serum TSH level and normal free thyroid hormone levels. Patients with normal levels of TSH and free thyroid hormones were deemed as II. The normal ranges of TSH, FT4, and FT3 were 0.27 to 4.2, 0.93 to 1.71, and 0.85 to 2.02 mU/L, respectively. Antimicrosomal antibody titers were analyzed in 84 patients.

### 2.2 US evaluation

**Figure 1:**
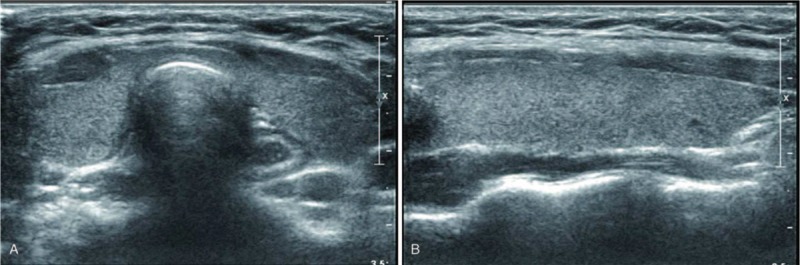
(A) Transverse and (B) longitudinal ultrasonographic images of Grade 1.

**Figure 2:**
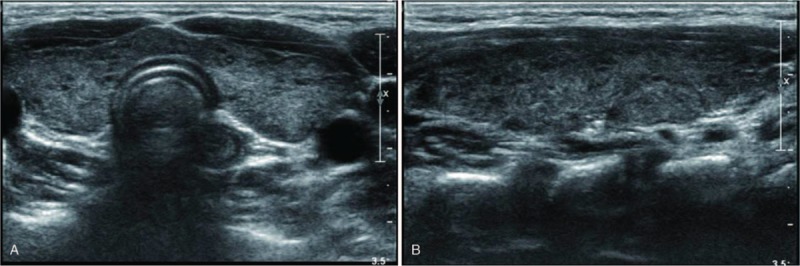
(A) Transverse and (B) longitudinal ultrasonographic images of Grade 2.

**Figure 3:**
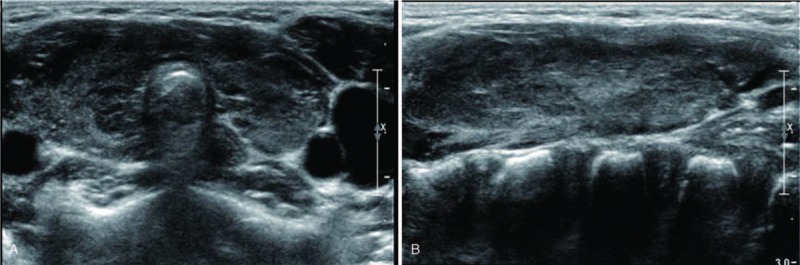
(A) Transverse and (B) longitudinal ultrasonographic images of Grade 3.

**Figure 4:**
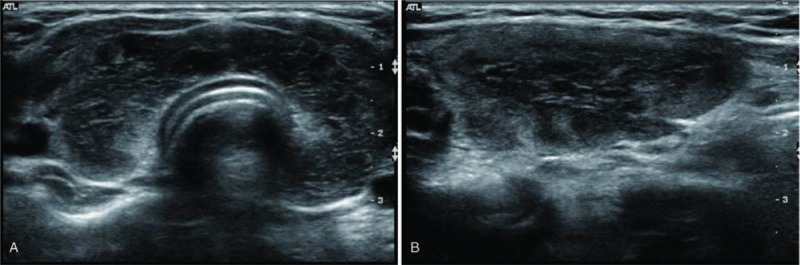
(A) Transverse and (B) longitudinal ultrasonographic images of Grade 4.

US and color Doppler examinations were performed using a 5 to 12 MHz linear array transducer (IU22 US or HDI 5000; Philips, Bothell, WA; or Aplio 500; Canon Medical Systems, Nasushiobara, Japan). One of 3 radiologists with 5, 8, and 25 years of experience in thyroid imaging performed the US examination. The US findings were retrospectively reviewed by radiologists with 5 and 25 years of experience in thyroid imaging and consensus was achieved; the reviewers were blinded to the hormonal status. The US patterns were classified into 4 grades to compare the thyroid echogenicity and anterior strap muscle echogenicity, which was used as a reference: Grade 1 (G1): diffusely enlarged gland with a normoechoic US pattern (similar to normal tissue, and to the submandibular gland, hyperechoic to the anterior strap muscle) (Fig. 1A and B); G2: multiple hypoechoic foci or patches scattered throughout an otherwise normal echogenic gland and involving <1/3 of the gland (Fig. 2A and B); G3: an enlarged gland with diffuse but mild hypoechogenicity (more hypoechoic than normal thyroid tissue, but hyperechoic compared to the anterior strap muscle) (Fig. 3A and B); and G4: an enlarged gland with diffuse, marked hypoechogenicity (less than or equal to the anterior strap muscle) (Fig. 4A and B).

### 2.3 Statistical analyses

Data are reported as means ± standard deviation for continuous variables and frequency (percentage) for categorical variables. To compare clinical characteristics between groups, the chi-square (*χ*^2^) or Fisher exact test were used for the categorical variables. For the continuous variables, Student *t* test was used to compare 2 groups and the Kruskal–Wallis test was used to compare the 4 levels of US grade after testing for normality and equivalent variance. To determine interobserver reproducibility, both observers performed their evaluations of the ultrasonographic findings independently. Interobserver and intraobserver agreement were calculated using the Cohen kappa test. A 2-tailed *P*-value of <.05 was considered statistically significant. All statistical analyses were performed using R software (ver. 3.3.2; R Foundation for Statistical Computing, Vienna, Austria).

## 3 Results

### 3.1 Demographics

The mean age of the patients was 11.1 ± 3.7 years (range 4–18 years). There were 12 (14.0%) males and 74 (86.0%) females. Levels of microsomal antibodies were evaluated in 84 patients. Comparisons of the total patients and patients with microsomal Ab were tabulated, and no significant difference was found for age (*P* = .887) and sex (*P* = 1).

### 3.2 Ultrasonographic grading versus functional status of the thyroid gland

Table 1 summarizes the relationship between US grade and functional thyroid status. There were 27 (31.4%), 22 (25.6%), 19 (22.1%) ,and 18 (20.9%) patients classified into groups G1 to G4 groups, respectively. G1 and G2 groups included 1 (1/27; 3.7%) and 2 (2/22; 9.1%) cases of overt hypothyroidism, 15 (15/27; 55.6%) and 13 (13/22; 59.1%) of subclinical hypothyroidism, and 11 (11/27; 40.7%) and 5 (5/22; 22.7%) of euthyroidism, respectively. In addition, 2 (2/22; 9.1%) patients in G2 group had hyperthyroidism. In G3 group, there were 15 (15/19; 78.9%) patients with overt hypothyroidism, 3 (3/19; 15.8%) with hyperthyroidism, and 1 (1/19; 5.3%) with subclinical hypothyroidism. All of the patients in G4 group had overt hypothyroidism. Among the patients with overt hypothyroidism, 91.7% (33/36) were G3 or G4 groups. By contrast, 97.8% (44/45) of the patients with subclinical hypothyroidism or euthyroidism were G1 or G2 groups, as initially evaluated via US. According to our results, patients with higher US grades had decreased thyroid function (*P* < .001). Thyroid function differed significantly among the 4 US groups (*P* < .001) and patients with higher grades had reduced thyroid function. The US grade increased with the TSH titer (*P* < .001), while the FT4 level significantly decreased with increasing US grade (*P* < .001). The microsomal antibody titer was not significantly related to US echogenicity. Interobserver agreement was 0.74 and intraobserver agreement was 0.85.

**Table 1:**
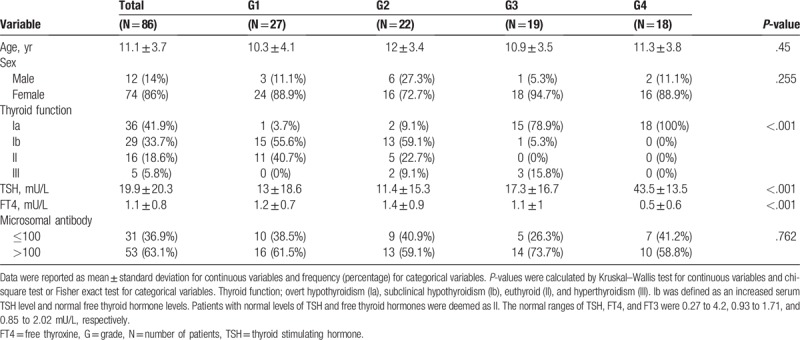
Baseline and clinical characteristics per sonographic grading.

## 4 Discussion

HT, also known as lymphocytic thyroiditis, is a goitrous form of AIT, and the most common thyroid disorder in children and adolescents. HT is characterized by diffuse lymphocyte and plasma cell infiltration, fibrous replacement, and eventual atrophy of the parenchyma.^[1–3]^ HT is the most common cause of hypothyroidism in iodine-sufficient countries, although the incidence varies with geographic location. Overt hypothyroidism is a common but potentially fatal medical condition evident in approximately 10% of HT patients; onset is insidious and the condition may not become clinically apparent until the full range of symptoms develop.^[14]^ While subclinical and overt hypothyroidism share similar etiologies, the symptoms of the former are nonspecific and signs of hypothyroidism are typically absent.^[15]^ Consequently, a diagnosis of subclinical hypothyroidism depends solely on thyroid function tests. Although subclinical hypothyroidism is a somewhat benign abnormality compared to other thyroid diseases, its absence of specific, related symptoms, and the difficulty in determining whether treatment is required, remain as challenges to both physicians and patients. Problems of diagnosing and treating subclinical hypothyroidism are becoming progressively more important as human life expectancy increases, because thyroid dysfunction, including subclinical hypothyroidism, is found more often in the elderly.^[11,13–15]^ Subclinical hypothyroid patients with underlying thyroid disease have a greater risk of progression to overt hypothyroidism,^[12]^ which is associated with adverse effects on lipid metabolism and cardiovascular function. However, predicting disease progression and assessing the risk of progression to a more severe state of thyroid dysfunction are challenging. There is no consensus regarding the clinical parameters associated with the prognosis for this mild thyroid dysfunction, although elevated serum TPOAb and decreased or heterogeneous echogenicity (diffuse thyroid disease) on US are often observed.^[16]^

Many studies have examined US in thyroiditis and its association with echogenicity, mainly hypoechogenicity, of the thyroid gland and thyroid function. The association between decreased echogenicity of the thyroid gland when examined with US and overt hypothyroidism is well known in HT.^[8,10]^ A change in echogenicity is also seen in subclinical hypothyroidism.^[4,5]^ However, previous studies included only adults or adults and adolescents; the present study evaluated this association in a pediatric population.

We found that pediatric and adolescent HT patients also show a significant correlation between thyroid US echogenicity and thyroid function, not only in overt hypothyroidism but also in subclinical and euthyroidism. There was also a strong association between decreased echogenicity and TSH level in pediatric and adolescent patients. According to Vejbjerg et al,^[5]^ younger patients show a stronger association between decreased echogenicity and a higher TSH, and the relationship is stronger still when the changes are recent. Decreased echogenicity in thyroid US scans in patients with elevated TSH might be an early sign of thyroid failure.^[4,9]^ Thyroid US in subclinical hypothyroidism has significant predictive value in terms of treatment outcome, comparable to other well-known prognostic parameters such as microsomal antibodies and high TSH levels.^[12]^ In our study, there was no significant association between echogenicity and microsomal antibody level. Of the few studies that investigated the relationship between thyroglobulin antibody and hypoechogenicity, none have found any such association.^[10,17]^ Thyroid US in conjunction with a microsomal antibody assay during initial assessment of a patient with subclinical hypothyroidism appear to be more helpful than microsomal antibody alone in predicting the clinical course and establishing a treatment plan, particularly in cases with mildly elevated TSH levels.^[13]^ Thyroid echogenicity can be used to predict thyroid failure.

There were some limitations to this study; it used a retrospective design and included a relatively small number of participants who underwent US examinations at a single institution. Therefore, a larger series should be conducted to validate our results. Furthermore, we did not conduct a follow-up US of consecutive patients. A previous study examined apparently healthy subjects with a 3-year follow-up and found that none of those who developed thyroid dysfunction had normal echogenicity in thyroid US scans at the time of inclusion.^[9]^ Another study demonstrated that all patients who developed hypothyroidism during follow-up had decreased echogenicity at baseline.^[18]^ This suggests that hypoechogenicity is an early marker of hypothyroidism. Further studies that follow up on changes in thyroid echogenicity and function are needed. Another limitation was that US is a subjective measure based on the experience and skills of the sonographer. Although we achieved consensus among 3 experienced radiologists, the reliability of the results is open to dispute. There has been some effort to quantify sonographic echogenicity,^[19,20]^ but it is not used widely. Therefore, subjective sonographic findings are still important.

## 5 Conclusion

In our pediatric and adolescent HT patients, there was an association between decreased echogenicity and thyroid dysfunction and high TSH levels, even in subjects with normal thyroid function, or subclinical or overt hypothyroidism. This suggests that US can be useful for supplementing biochemistry assays in the evaluation of thyroid status. Further investigations with follow-up studies are needed to evaluate the usefulness of US to predict the progression of abnormal thyroid function.

## Acknowledgments

We are grateful to Bora Lee (Research Director, Research & Development Center, Rexsoft Co., Ltd. Researcher of Public Health Science, Graduate School of Public Health, Seoul National University) for the statistical analysis.

## Author contributions

**Conceptualization:** Hyun Sook Hong.

**Data curation:** Hyun Sook Hong, Ji Ye Lee.

**Formal analysis:** Hyun Sook Hong, Sun Hye Jeong, Ji Ye Lee.

**Investigation:** Hyun Sook Hong, Sun Hye Jeong, Ji Ye Lee.

**Methodology:** Hyun Sook Hong, Sun Hye Jeong, Ji Ye Lee.

**Project administration:** Hyun Sook Hong.

**Resources:** Hyun Sook Hong, Sun Hye Jeong, Ji Ye Lee.

**Validation:** Hyun Sook Hong, Ji Ye Lee.

**Visualization:** Hyun Sook Hong.

**Writing - Original Draft:** Hyun Sook Hong, Sun Hye Jeong.

**Writing - Review and Editing:** Hyun Sook Hong, Ji Ye Lee.

Hyun Sook Hong orcid: 0000-0003-3210-9740.
